# Temporal Changes in Population Structure of a Marine Planktonic Diatom

**DOI:** 10.1371/journal.pone.0114984

**Published:** 2014-12-15

**Authors:** Sylvie V. M. Tesson, Marina Montresor, Gabriele Procaccini, Wiebe H. C. F. Kooistra

**Affiliations:** Stazione Zoologica Anton Dohrn, Villa Comunale, 80121 Naples, Italy; University of Sydney, Australia

## Abstract

A prevailing question in phytoplankton research addresses changes of genetic diversity in the face of huge population sizes and apparently unlimited dispersal capabilities. We investigated population genetic structure of the pennate planktonic marine diatom *Pseudo-nitzschia multistriata* at the LTER station MareChiara in the Gulf of Naples (Italy) over four consecutive years and explored possible changes over seasons and from year to year. A total of 525 strains were genotyped using seven microsatellite markers, for a genotypic diversity of 75.05%, comparable to that found in other *Pseudo-nitzschia* species. Evidence from Bayesian clustering analysis (BA) identified two genetically distinct clusters, here interpreted as populations, and several strains that could not be assigned with ≥90% probability to either population, here interpreted as putative hybrids. Principal Component Analysis (PCA) recovered these two clusters in distinct clouds with most of the putative hybrids located in-between. Relative proportions of the two populations and the putative hybrids remained similar within years, but changed radically between 2008 and 2009 and between 2010 and 2011, when the 2008-population apparently became the dominant one again. Strains from the two populations are inter-fertile, and so is their offspring. Inclusion of genotypes of parental strains and their offspring shows that the majority of the latter could not be assigned to any of the two parental populations. Therefore, field strains classified by BA as the putative hybrids could be biological hybrids. We hypothesize that *P. multistriata* population dynamics in the Gulf of Naples follows a meta-population-like model, including establishment of populations by cell inocula at the beginning of each growth season and remixing and dispersal governed by moving and mildly turbulent water masses.

## Introduction

Marine planktonic organisms can grow extremely fast. Such fast growth, sustained by plentiful resources and temporally relaxed predation pressure, can lead to episodic, rapid and vast increases in their population sizes. The huge numbers of individuals and the moving and mixing water masses they inhabit are expected to foster large-scale population genetic homogeneity. Yet, a series of recent studies demonstrated that geographic structuring can occur in marine planktonic organisms [1]–[3]. In the case of the jellyfish *Aurelia,* trans-oceanic populations exist genetically in isolation-by-distance because the restricted lifespan of its planktonic medusa-stage prohibits gene flow across such extensive tracts of ocean [1].

Unicellular phytoplankton species usually show high genotypic diversity and in cases where genetically distinct populations are observed, they are often correlated with hydrographic or geographic features [1], [4]–[10]. Marine eukaryotic microalgae grow by means of mitotic division, but in contrast to daughter cells in macrophytes and animals, microalgal daughter cells disconnect and drift apart in their mildly turbulent environment, thus forming widely distributed clones. Episodic sexual reproduction in a population composed of large numbers of clones generates huge numbers of F1 cells with distinct genotypes, each of which in its turn can form a clone [11]. Therefore, the likelihood of sampling multiple individuals belonging to the same clone in a large phytoplankton population is very small, given the sample sizes normally deployed in population genetic studies [12].

Although the emergence of genetic differentiation without geographic barriers remains highly controversial, speciation can occur in sympatry [13] that is, if populations reproduce in distinct temporal windows, and/or have distinct ecological niches [14]–[15]. In phytoplankton, Casteleyn *et al*. [3] demonstrated that strains of the diatom *Pseudo-nitzschia pungens* established from cells collected in Belgian, Danish and Irish waters grouped into two genetically distinct, but apparently sympatric populations. Such genetic distinctness could merely be temporal, for instance resulting from contemporary establishment of founder populations from distinct sources, to be homogenized if sexual reproduction can still occur amongst them. In fact, marine habitats are among the most heavily invaded systems on Earth [16] and this is not necessarily restricted to invasions of alien species, but also to alien populations of resident species (e.g., [17]). Alternatively, mate preference and/or slightly offset bloom windows may keep these sympatric populations genetically segregated. If this is the case, then subtly different performance, e.g., different growth rates and environmentally governed differential mating success, could explain radical shifts in their proportions from one growth season to the next.

Few studies have addressed the structure of planktonic microalgal species over a temporal scale. A considerable genetic differentiation was detected over two consecutive years for the dinoflagellate *Alexandrium fundyense* in a coastal pond, where different populations were detected even amongst samples collected after 7 days. These highly diverse and dynamic patterns contrast with the constant genetic structure of the diatom *Skeletonema marinoi,* where samples composed of strains resulting from the germination of up to 100-years old resting stages collected from laminated sediment cores were found to belong to the same population as samples composed of strains obtained from the present-day plankton from the same site [18].

**Figure 1:**
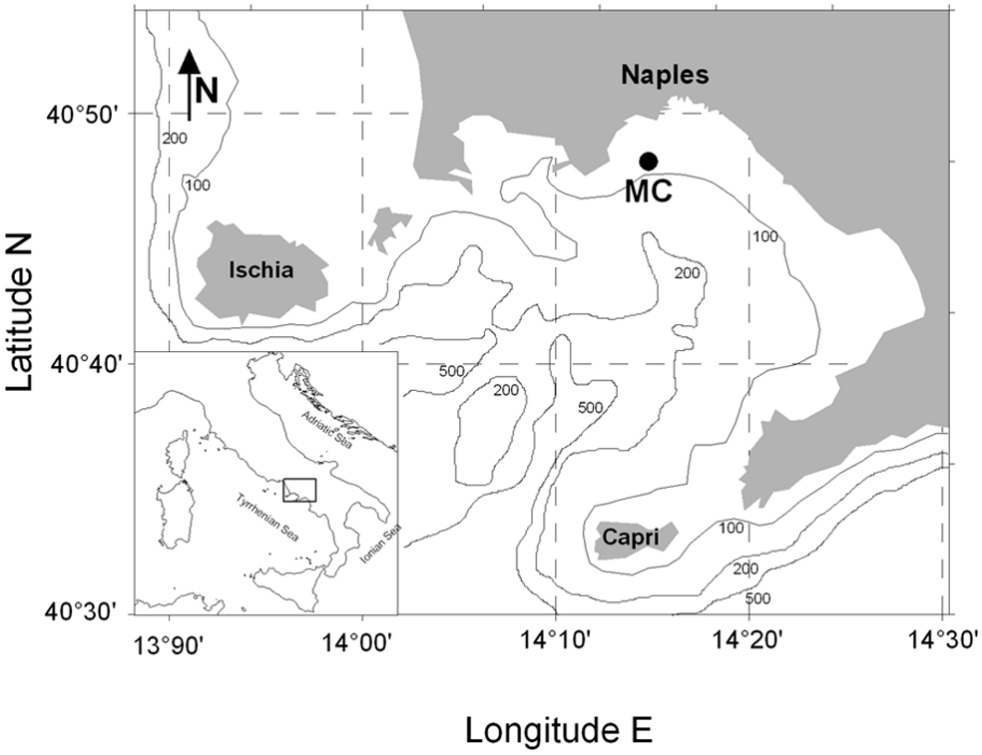
The Gulf of Naples (GoN) and the location of the Long Term Ecological Research station MareChiara (LTER-MC; 40°48.5′N, 14°15′E).

In the present study we explore population genetic structure of the planktonic, chain-forming diatom *Pseudo-nitzschia multistriata* at the Long Term Ecological Research station MareChiara (LTER-MC) in the Gulf of Naples (GoN, Tyrrhenian Sea, Mediterranean Sea, **Fig. 1**). The species has been recorded here from summer until late autumn of every year since its first appearance in the GoN, in 1996 [19]–[21].

*Pseudo-nitzschia multistriata,* as almost all diatoms, possesses rigid siliceous cell wall elements. During cell division each daughter cell inherits a parental cell wall element and makes a new one that fits precisely inside the parental one. Hence average cell size diminishes with ongoing mitotic division and the variance around the average increases. Populations of clonal cell lines escape from this miniaturization trap through sexual reproduction, upon which large cells (82–72 µm long) are formed within the zygote, thus reestablishing the initial large cell size. Yet, cells can be sexualized only within a restricted window of cell sizes [22]. A life cycle model of *P. multistriata* in the GoN by D’Alelio *et al.* [21] predicts that sexual reproduction in this species occurs once every two years. If the model is correct, then only six or seven sexual reproduction events have occurred between its first appearance in the GoN in 1996 and the onset of our population genetic sampling, in 2008.

To investigate intraspecific genetic diversity and population genetic structure of *P. multistriata*, we obtained seven polymorphic microsatellite markers [23] and used these to genotype strains generated from single cells, or clonal chains of cells, isolated from 22 net samples obtained during the species’ seasonal appearance at the LTER-MC station over four consecutive years (September 2008 to July 2011). The genotype data were analyzed utilizing Bayesian clustering software to assess the most probable number of genetically distinct populations. Evidence emerged for the occurrence of multiple populations in the GoN, and therefore we assessed if these were separated in time, i.e., by sampling date, season or year, and if there were signs of them merging, e.g., by the increasing proportion of hybrids. In order to support the idea that sampled strains assigned as putative hybrids resulted from crossings between strains assigned to different populations, we included the genotypes of F1 offspring strains and their parental lineages, generated in Tesson *et al*. [11] in our analysis.

## Materials and Methods

### Ethics Statement

The present study on the planktonic diatom *Pseudo-nitzschia multistriata* was performed in accordance with the Italian laws and did not investigate endangered or protected species. No specific permissions were required to collect surface phytoplankton samples at the LTER-MC (40°48.5′N, 14°15′E, **Fig. 1**) in the Gulf of Naples.

### Sampling site

The Gulf of Naples (GoN; **Fig. 1**) is an embayment open to the Tyrrhenian Sea (Western Mediterranean) and connected to the nearby Gulfs of Gaeta and Salerno (North-west and South-east, respectively) by slow seasonal cyclonic and anti-cyclonic eddies and offshore currents [24]. The sampling site, the LTER-MC, is located two miles offshore in the Gulf and is characterized by a surface temperature between 14°C in February–March and 26°C in August, a salinity of 37.5–38.0 (±0.1) and the presence of a seasonal thermocline from April to September [19].

### Cell collection, identification and culture maintenance

Surface phytoplankton samples were collected weekly using a rosette sampler equipped with Niskin bottles. Water samples for cell counts were fixed with neutralized formaldehyde at a final concentration of 0.8%. Depending on the total phytoplankton cell abundance of the samples, between 1 and 50 ml were allowed to settle in combined sedimentation chambers and cell concentration was estimated at 400x magnification [25].

For the isolation of living cells and chains, phytoplankton samples were collected with a standard phytoplankton net (20 µm mesh size). Cells and chains of *Pseudo-nitzschia multistriata* were isolated from 21 net samples collected between September 2008 and September 2010, and from a single net sample taken July 2011 (**Table 1**). Isolated cells were incubated each in 2 ml f/2 filtered medium at 22°C, *ca.* 80 µmol photons·m^−2^·s^−1^, and a photoperiod 12L:12D hour (Light : Dark). After a few days, isolation success and purity of the cultured strains were checked using an inverted light microscope; average cell size was determined by measuring cell length over the apical axis of at least 5 cells per strain at 200x magnification. After one week of growth, the obtained strains were transferred into culture bottles (Corning Flask, Corning Inc., NY, USA) containing 25 mL of f/2 medium to reach a concentration of about 10^3^ cell·ml^−1^. A total of about 1100 single cells or short chains were isolated, and of these, 735 (66.8%) were grown successfully in culture. Strain isolation success was comparable among sampling dates.

**Table 1:** Genetic diversity of the strains of *Pseudo-nitzschia multistriata* sampled within the 22 field samples collected at the Long Term Ecological Research station MareChiara.

Sample Nr.	Sample date	N	G	G/N	A/l	Na	Na7	SE	PA	He	Ho	F_IS_ ± SE
1	170908	21	16	76.2	4.43	31	22.830	0.077	2	0.566	0.687	−0.19±0.26
2	230908	53	40	75.5	5.57	39	21.733	0.076	2	0.536	0.717	−0.31±0.20
3	300908	44	39	88.6	6.57	46	23.314	0.088	5	0.552	0.750	−0.31±0.19
4	141008	18	13	72.2	3.43	24	19.859	0.046	0	0.521	0.730	−0.38±0.24
5	041108	7	6	85.7	3.29	23	-	-	0	0.509	0.714	−0.39±0.14
6	111108	7	5	71.4	2.86	20	-	-	0	0.501	0.735	−0.40±0.27
7	181108	7	6	85.7	3.57	25	-	-	1	0.539	0.673	−0.25±0.11
8	300609	24	24	100.0	4.86	34	24.678	0.070	3	0.535	0.524	0.03±0.10
9	070709	35	33	94.3	4.71	33	23.034	0.054	1	0.527	0.531	0.09±0.13
10	140709	18	14	77.8	4.14	29	22.879	0.065	2	0.559	0.587	0.02±0.25
11	220909	8	8	100.0	3.14	22	21.627	0.015	0	0.551	0.607	−0.07±0.13
12	290909	57	39	68.4	5.43	38	22.554	0.085	1	0.544	0.624	−0.05±0.21
13	061009	24	14	58.3	4.71	33	22.711	0.113	0	0.540	0.601	−0.03±0.25
14	201009	14	10	71.4	3.00	21	18.568	0.053	0	0.454	0.510	−0.09±0.16
15	271009	13	6	46.2	3.14	22	18.892	0.045	1	0.464	0.626	−0.25±0.20
16	150610	9	8	88.9	3.29	23	21.845	0.021	0	0.477	0.571	−0.23±0.12
17	060710	14	11	78.6	3.57	25	20.519	0.053	0	0.491	0.561	−0.19±0.15
18	100810	33	33	100.0	5.71	40	26.834	0.074	14	0.609	0.628	−0.04±0.13
19	070910	48	47	97.9	6.57	46	25.157	0.076	4	0.578	0.598	−0.03±0.12
20	140910	32	29	90.6	4.29	30	21.847	0.070	0	0.571	0.554	0.05±0.16
21	210910	26	18	69.2	3.86	27	21.544	0.060	2	0.576	0.484	0.13±0.22
22	100711	13	13	100.0	4.71	33	25.332	0.043	2	0.553	0.593	0.01±0.21

Sampling dates are expressed as DDMMYY. Included in the table are the number of strains analyzed (N), the number of genotypes (G), the genotypic diversity (G/N, in %), the average number of alleles per locus (A/l), the total number of alleles (Na), the average number of alleles when resampled 1000 times for N = 7 as smallest sample size (Na7) and associated Standard Error (SE), the number of private alleles (PA), the expected (He) and observed (Ho) heterozygosity, and the fixation index (F_IS_ ± SE) over the seven microsatellite loci.

### DNA extraction and strain genotyping using microsatellite markers

Genomic DNA of *P. multistriata* was extracted as described in Tesson *et al.* [23]. DNA-purity and quantity were assessed using the NanoDrop Spectrophotometer (ND-1000, NanoDrop, Wilmington, DE, USA) and agarose gel electrophoresis (1.5%). Pure DNA was diluted to 25 ng·µl^−1^ and 2 µl of the dilution was added to a well in a 96-well-plate containing the polymerase chain reaction (PCR) mix [23]. Seven microsatellite markers (*PNm1, PNm2, PNm3, PNm5, PNm6, PNm7,* and *PNm16*) were amplified in multiplexes by PCR, purified and combined for microsatellite identification as described in Tesson *et al.* [23] using forward labeled primers. Microsatellite reactions were prepared in automation with a robotic station Biomek FX (Beckman Coulter, Fullerton, CA) and analyzed on an Automated Capillary Electrophoresis Sequencer 3730 DNA Analyzer (Life technologies, 5791 Van Allen Way Carlsbad, CA 92008 USA). Electropherogram analysis was performed using Peak Scanner (version 1.0, Applied Biosystems). Peak assignment was refined manually in order to avoid scoring mistakes. In the few cases where stutter bands were present and interpretations were not univocal, samples were re-run up to three times to confirm scoring.

### Microsatellite data analysis

Micro-Checker (version 2.2.3 [26]) was applied to calculate the frequency of null-alleles (i.e., non-amplification of alleles) for each of the loci under the assumption that the loci are in HWE (**S1 Table**). A Brookfield-1 estimation was applied following recommendation by the program manual. Tests of presence of stutter bands due to slight changes in microsatellite length and low efficiency of large alleles amplification (large allele dropout) in PCR were performed as described in the manual (**S1 Table**).

Average number of alleles per locus, expected and observed heterozygosity, the inbreeding coefficient F_IS_, the fixation index F_ST_, and the G_ST_ index of diversity across population were calculated for groups of strains using GenAlEx (version 6 [27]). F_IS_ is defined here as the proportion of the genetic variance in a group of strains contained in a sample. A significant positive F_IS_ implies a significant inbreeding/homozygote over-representation, whereas a significant negative value, significant outbreeding/heterozygote over-representation; F_ST_, is defined here as the proportion of the total genetic variance contained in a group of strains relative to the total genetic variance among all of the strains; G_ST_, is a relative measures of F_ST_ that takes into the account differences in sample size and the amount of genetic variation among populations versus all populations.

Allelic richness was re-calculated for each sample using GenClone [28]. In order to compare groups (i.e., samples, sampling years) with different numbers of strains, allelic richness was estimated in each group, with a resampling procedure, performing 1000 permutations for all possible numbers of sampling units (from 1 to N). Values obtained for the same number of samples were taken for comparison. Genotypic diversity (G/N) within samples was estimated using Gimlet (version 1.3.3 [29]).

The number of genetically distinct populations and their occurrence through time were inferred using a Bayesian cluster analysis performed with Structure (version 2.1 [30]) The number of clusters (K; populations) was estimated following Evanno *et al*. [31] using the web-based program Structure Harvester [32]. Population structure was obtained applying a burn-in of 15,000 iterations, with 75,000 MCMC repetitions from K = 1 to K = 23. The parameters used to infer genetic structure were an ancestry model with admixture, along with an independent frequency model (infer λ estimated to 0.1414). The LOCPRIOR assumption was not applied. The Assignment Probability (AP) threshold for a strain to a population assigned by Structure was set at 90%. A separate Structure analysis with the same settings was performed also including the genotypes of a set of parental strains and their F1 progeny obtained from Tesson *et al.* [11]. The existence of a bottleneck effect in the two populations and in consecutive years was tested using the Wilcoxon test implemented in the software Bottleneck [33], after 1000 iterations and hypothesizing the two-phased model (TPM) of mutation.

Genetix (version 4.05.2 [34]) was used to identify linkage disequilibrium (10,000 permutations and a significance level of 0.05) within the whole data set. Factorial Correspondence Analysis was also performed with Genetix, using genotypes assigned to populations as inferred from Structure and those not assigned to any of them. Principal Component Analysis (PCA) based on single strains as well as on groups of strains was performed using GenAlEx and the pairwise matrix of Nei genetic distance. Statistical analysis and graphical representation of *P. multistriata* abundance and frustule size distribution - of cells observed in formalin fixed field samples as well as of cells from which the genotyped strains were raised - were performed using R [35].

## Results

**Figure 2:**
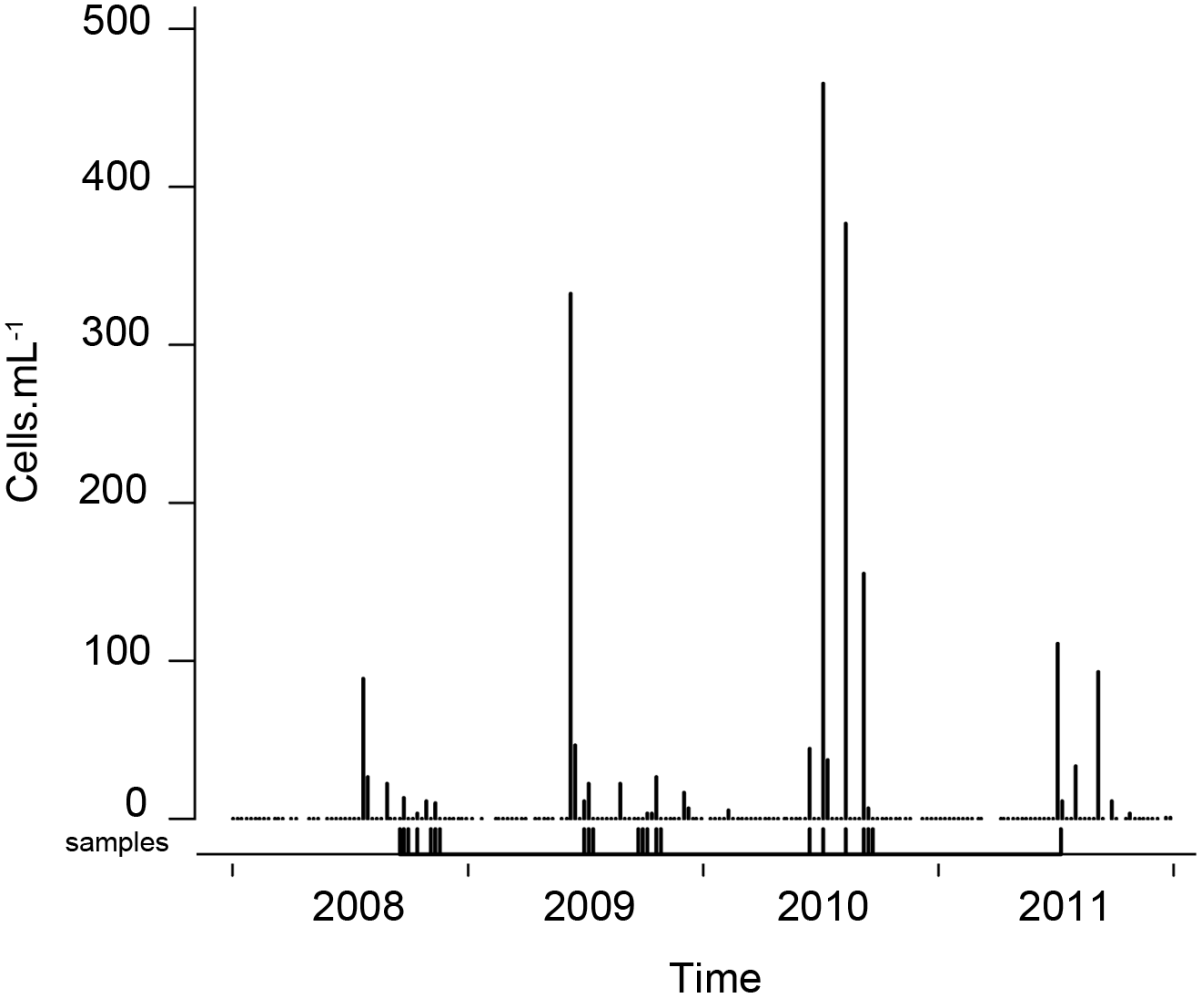
Cell concentration of *Pseudo-nitzschia multistriata* in the surface samples collected at LTER-MC from January 2008 to December 2011. All LTER-MC samples are indicated with dots along the x-axis. The LTER-MC samples from which *P. multistriata* cells have been isolated for genotyping are indicated with small vertical bars under the sample dots (for sample numbers see Table 1).

**Figure 3:**
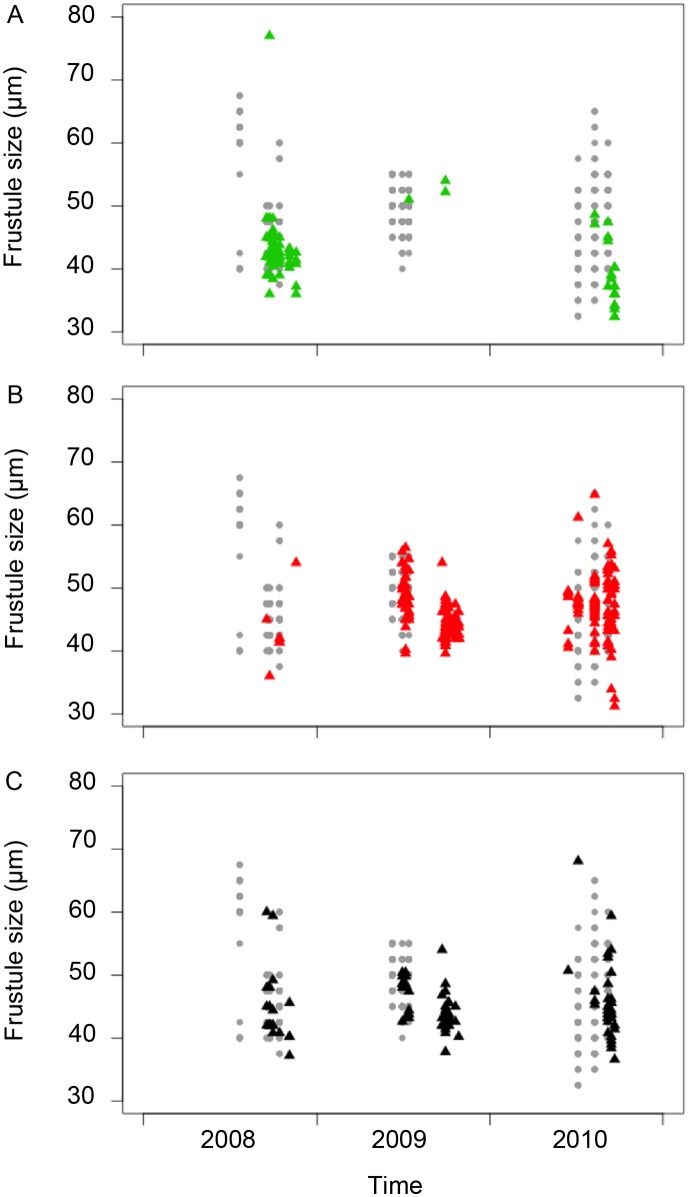
Cell length of *Pseudo-nitzschia multistriata* strains assigned to A) POP_A (green), B) POP_B (red), and C) putative hybrids (black), as determined by Structure. The grey dots in the background represent the cell size classes recorded in the natural environment.

*Pseudo-nitzschia multistriata* was detected in the surface layer at the LTER-MC from June/July until October/November of the four sampled years (2008–2011), with cell abundances up to 465×10^3^ cells l^−1^ (**Fig. 2**). A total of 525 strains out of the 735 successfully grown in culture were successfully genotyped from 22 of the net-samples in which the species was present (157 strains in samples 1–7 in 2008; 193 in samples 8–15 in 2009; 162 in samples 16–21 in 2010, and 13 in sample 22 in 2011; **Table 1**). Twelve of these strains belonged to the frustule size class ≥55 µm, in which sexual reproduction cannot be induced, whereas the remainder belonged to the 55–30 µm size-interval, i.e. the size window in which sex can be induced (**Fig. 3**). We assessed cell size distribution of *P. multistriata* in a limited number of samples collected between 2008 and 2010 (**Fig. 3**). The cell size ranges observed in these natural samples matched those observed among the cells from these samples (**Fig. 3**).

### Genetic diversity within samples and sample years

The 525 strains generated a clearly readable microsatellite fingerprint without any apparently missing values for any of the seven loci (**S2 Table**). All loci were polymorphic in all of the 22 samples, except locus *PNm3*, which was monomorphic in three samples (data not shown). The observed heterozygosity (Ho) was higher than the expected (He) – and often markedly so - in all samples except 8, 20 and 21. The inbreeding coefficient F_IS_ was significantly negative for almost all of the samples from 2008 (samples 2–7) and for samples 15 and 16, implying significant outbreeding. The average inbreeding coefficient (F_IS_) over all 22 sampling dates was negative (−0.131±0.039) indicating outbreeding and overall heterozygosity excess (**Table 1**).

The number of alleles per locus ranged from 7 to 21. All loci, except *PNm1* and *PNm5,* showed heterozygosity excess. No allele-drop out was detected in any of the loci (**S1 Table**). Only two loci (*PNm2-PNm5*) were in linkage disequilibrium within the whole data set (data not shown).

The strains exhibited 394 distinct genotypes (G). The genotypic diversity (G/N) ranged from 46.2% to 100% across the individual samples (**Table 1**), from 66.2% for 2008, to 87.0% for 2010, with an average of 75.5% over all 22 samples (**Table 2**). No clear relationship was detected between sample date and genotypic diversity, also because sample size varied markedly among sample dates.

**Table 2:** Genetic diversity of *Pseudo-nitzschia multistriata* per year of sampling (2008–2011).

Samples	Sample year	N	G	G/N	A/l	Na	Na157	SE	PA	He	Ho	F_IS_ ± SE
1–7	2008	157	104	66.2	7.71	54	-	-	20	0.547	0.722	−0.285±0.20
8–15	2009	193	137	71.0	7.57	53	50.688	0.043	12	0.554	0.580	0.038±0.17
16–21	2010	162	141	87.0	8.28	58	57.567	0.022	33	0.610	0.572	0.045±0.13
22	2011	13	13	100.0	4.71	33	-*	-*	2	0.553	0.593	0.01±0.21

For sampling dates see Table 1.

Included in the table are the number of strains analyzed (N), the number of genotypes (G), the genotypic diversity (G/N, in %), the average number of alleles per locus (A/l), the total number of alleles (Na), the average number of alleles when resampled 1000 times for N = 157 as smallest sample size (Na157) and associated Standard Error (SE), the number of private alleles (PA), the expected (He) and observed (Ho) heterozygosity, and the fixation index (F_IS_ ± SE) over the seven microsatellite loci. Resampling for the smallest sampling size on the samples grouped per year was not performed for the 2011 sample (*).

The total number of alleles (Na) per sampling date, over the seven loci considered, ranged from 20 (sample 111108) to 46 (samples 300908 and 070910), with an average value of 30 alleles (±8.1) (**Table 1**). The normalized average number of alleles (Na7; resampling with N = 7, i.e., the smallest sample size) ranged between 18.568 (sample 201009) and 26.834 (sample 100810) (**Table 1**). The number of private alleles per sample (PA) ranged from 0 (at many sampling dates) to 14 (sample 100810) (**Table 1**). The total number of alleles (Na) per year was 54, 53, 58 and 33, in 2008, 2009, 2010 and 2011, respectively (**Table 2**). The number of private alleles (PA) per year ranged from 12 (2009) to 33 (2010), whereas the 13 strains sampled in 2011 showed two private alleles (**Table 2**).

### Population genetic patterning

**Figure 4:**
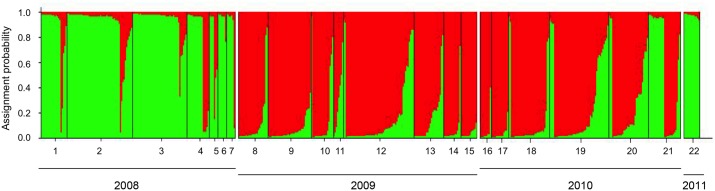
Genetic structure of the strains of *Pseudo-nitzschia multistriata* within each sample, as defined by Structure. Samples are presented in ranking order (1–22) along the x-axis. Each vertical bar represents one strain. The y-axis indicates the proportion of a strain’s genotype assigned by Structure to a population (i.e., K-cluster); the two populations identified by Structure are indicated POP_A (green), POP_B (red). Strains in each sample are ranked along the x-axis based on their assignment probabilities. Strains not assignable to any of the two populations above the 0.9-probability-level, are indicated to the right side of each sample. Samples have been grouped by year of collection: samples 1–7 in 2008, samples 8–15 in 2009, samples 16–21 in 2010 and sample 22 in 2011.

Results of the independent runs of K = 1 to 22 for assessing Bayesian population clustering as implemented in Structure, showed that the existence of two groups (K = 2; mean LnP(K) = −8683.8; SD LnP(K) = 1.78; ΔK = 206.7) was by far the most likely (**S1 Figure**). The inferred groups are denoted from here on as populations POP_A and POP_B (green and red, respectively in **Fig. 4**, **S2 Table**). Strains assigned to POP_A dominated in 2008 and in the single sample of 2011, whereas the ones assigned to POP_B dominated in 2009 and 2010 (**Table 3**). Since Structure results were obtained using the ancestry model with admixture, strains that could not be assigned to any of the two populations with a probability above the 90%-threshold were considered to be putative hybrids. These assigned hybrids constituted 15%, 27% and 24% of the strains sampled in 2008, 2009 and 2010, respectively, whereas no putative hybrids were assigned among the 2011-samples. The proportion of assigned hybrids differed significantly among years (p = 0.02861 χ^2^-test (χ^2^ = 7.11, df = 2)). The proportion of assigned hybrids did not differ significantly between 2008 and 2010 (p-value = 0.012), but both of these differed from that in 2009 (p-value>0.05).

**Table 3:** Genotypic diversity of the inferred *Pseudo-nitzschia multistriata* populations POP_A, POP_B and the putative hybrids as identified by Structure over the four years of study.

	N				G			G/N		
Year	POP_A	POP_B	Hybrids	Total	POP_A	POP_B	Hybrids	POP_A	POP_B	Hybrids
2008	126	7	24	157	75	5	24	59.5	*71.4*	*100*
2009	3	138	52	193	3	85	49	*100*	61.6	94.2
2010	22	101	39	162	12	92	37	54.6	91.1	94.9
2011	13	0	0	13	13	0	0	*100*	-	-
Total	164	246	115	525	103	182	110	62.8	74	95.7

Included in the table are the number of strains (N), the number of genotypes (G) and the genotypic diversity (G/N).

**Figure 5:**
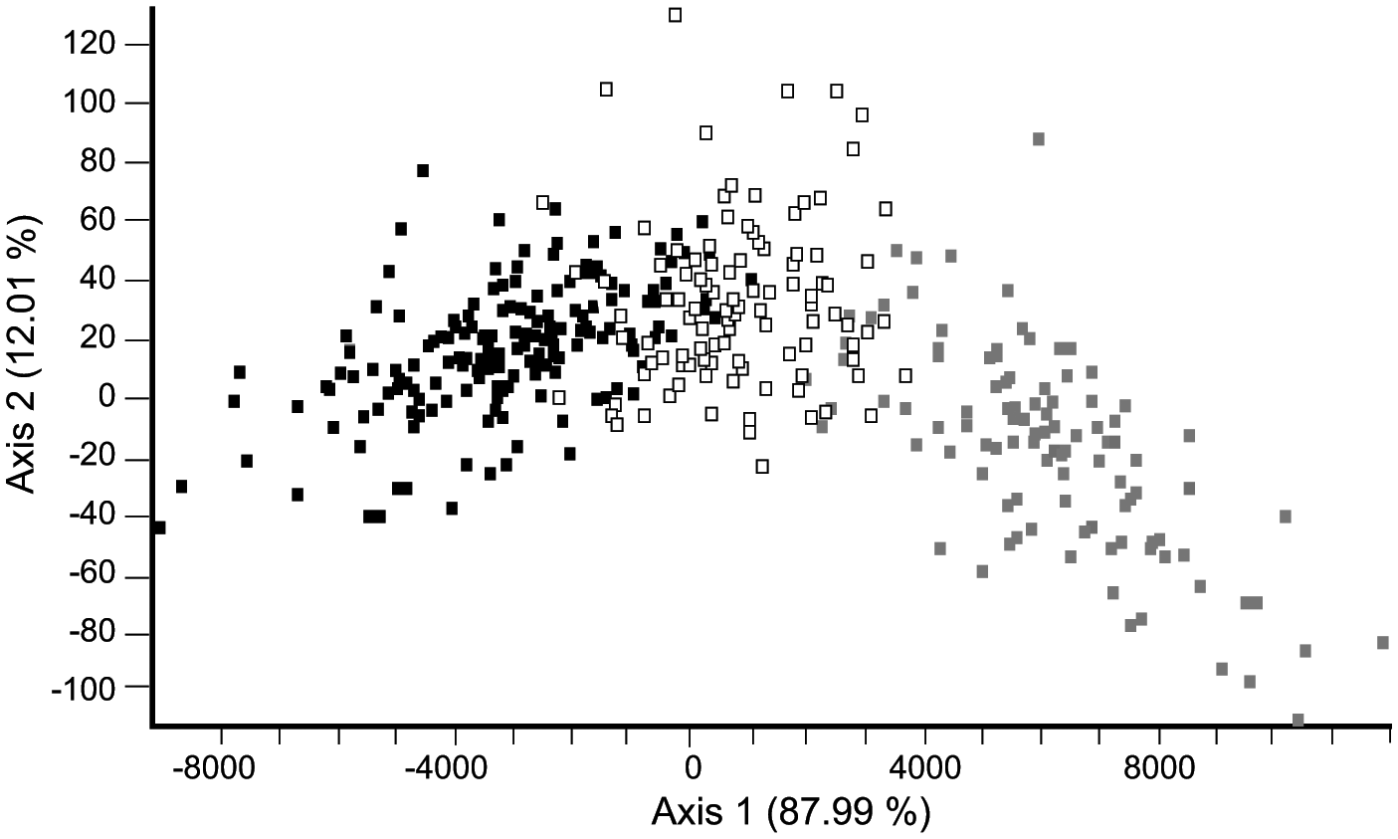
Placement of the individual *Pseudo-nitzschia multistriata* strains along the first two FCA axes (Genetix); specimens assigned by Structure to POP_A are indicated with grey squares, those to POP_B with black squares and those classified as putative hybrids (HYBR) with white squares.

**Figure 6:**
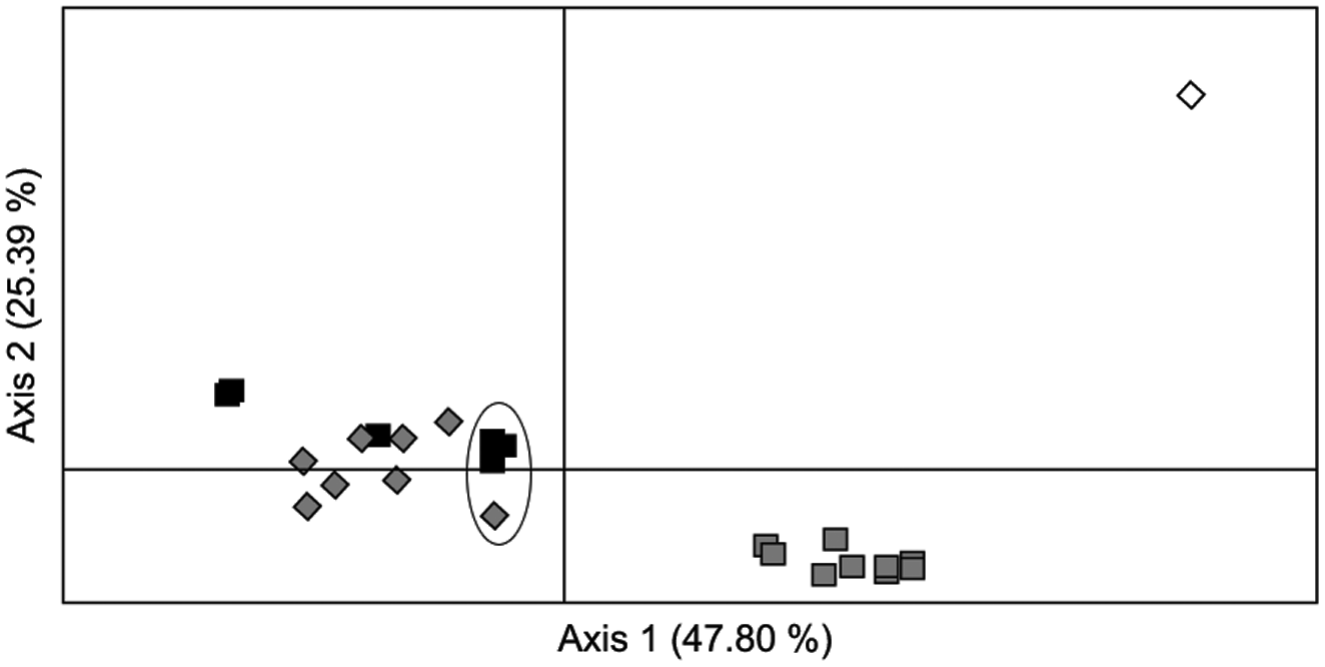
Placement of the 22 samples along the first two PCA axes (GEnALex); samples of *P. multistriata* gathered at LTER_MC in 2008 are indicated with grey squares; those in 2009 with grey diamonds, those in 2010 with black squares, and the one in 2011 with a white diamond. Samples 11 (2009), 19, 20, and 21 (2010), which showed a higher abundance of POP_B strains, but contained a minority of strains assigned to POP_A and others assigned as putative hybrids, are enclosed within an ellipse (see text). The two PCA axes capture 73.2% of the variability.

Factorial Correspondence Analysis (FCA) of the strains’ genotypes resulted in a plot in which all the strains were distributed along a stretched-out swarm without any apparent subdivision (**Fig. 5**). Strains assigned to POP_A grouped towards the right side, those assigned to POP_B towards the left side, and those classified as putative hybrids, lay in-between. PCA mapping of the 22 samples resulted in a plot (**Fig. 6**) in which the location of the samples corroborated the findings of Structure (**Fig. 4**). In addition, patterns not captured by Structure were uncovered. The 2008-samples, composed of strains mostly assigned to POP_A, clustered together on the lower right of **Fig. 6**, whereas sample 22 from 2011 (also POP_A) was also placed on the positive side of axis 1 but away from this cluster, to the top-far right. The 2009- and 2010-samples, composed of strains assigned mainly to POP_B, clustered on the left side. Samples 11 (2009), 19, 20 and 21 (2010) (enclosed in the ellipse in **Fig. 6**) were still dominated by POP_B strains, but, contained a minority of strains assigned to POP_A and others classified as putative hybrids, and were recovered on the right side of the POP_B-cluster, towards the POP_A-cluster.

### Genetic diversity of the two populations

If all the strains analyzed by Structure are taken together, then a total of 103 distinct genotypes were found among the 164 strains assigned to POP_A (G/N = 62.8%, **Table 3**), whereas 182 distinct genotypes were detected among the 246 strains assigned to POP_B (G/N = 74.0%). A total of 110 genotypes was identified (G/N = 95.7%) among the 115 strains that did not obtain an assignment probability ≥90% to POP_A or POP_B (**Table 3**). We classified these strains as putative hybrids. The genotypic diversity among POP_A strains was comparable between 2008 (G/N = 59.5%; N = 126) and 2010 (G/N = 54.6%; N = 22), while it was higher in the other two years (G/N = 100%), where only 3 individuals (in 2009) and 13 individuals (in 2011) were present. Differences in POP_A G/N values among years are not statistically comparable, since the population is represented by few strains in 2009, 2010 and 2011. The same accounts for POP_B in 2008 and 2011 (**Table 3**). The genotypic diversity among POP_B strains was significantly lower in 2009 (G/N = 61.6%; N = 138) than in 2010 (G/N = 91.1%; N = 101) (χ^2^ = 24.89, df = 1, p-value<0.01). Fifteen genotypes in POP_A and 16 in POP_B were shared by different strains within the same year, but not between different years (**Table 4**). Amongst the putative hybrids, five genotypes were shared; only one of these was present in different years (i.e. 2008 and 2010; **Table 4**). Neither of the two populations (POP_A and POP_B) was in HWE (p<0.05 and χ^2^-test >50). However, the fixation-index not significantly different from zero in both populations indicated random mating within populations (F_IS-_POP_A = −0.149±0.202 and F_IS-_POP_B = −0.083±0.090). POP_A and POP_B exhibited a similar effective number of alleles (POP_A: 18.86 alleles and POP_B: 18.55 alleles), and a similar number of private alleles (POP_A: 24 and POP_B: 23).

**Table 4:** Number of single, multiple and shared genotypes in the inferred populations, POP_A, POP_B and the putative hybrids, as identified by Structure over the four years of the present study.

	POP_A		POP_B		Hybrids	
Year	Ns	Gs	Ns	Gs	Ns	Gs
2008	64	13	3	1	1	1#
2009	0	0	64	11	5	2
2010	12	2	13	4	5	2#
2011	0	0	0	0	0	0
Total	76	15	80	16	11	5
Shared per year	0	0	0	0	2	1

Included in the table are the number of strains that shared a common genotype (Ns) and the number of shared genotypes (Gs). # indicates that genotypes are shared between different years.

*Pseudo-nitzschia multistriata* showed a marked seasonal cycle with high cell abundances restricted to a relatively short period. Results of the Wilcoxon test of each of the two populations sampled in consecutive years were negative, revealing no significant likelihood (p<0.05) for the existence of bottlenecks. Results were likewise after pooling all strains within single years.

### Genetic structure of the F1 samples resulting from crosses

**Figure 7:**
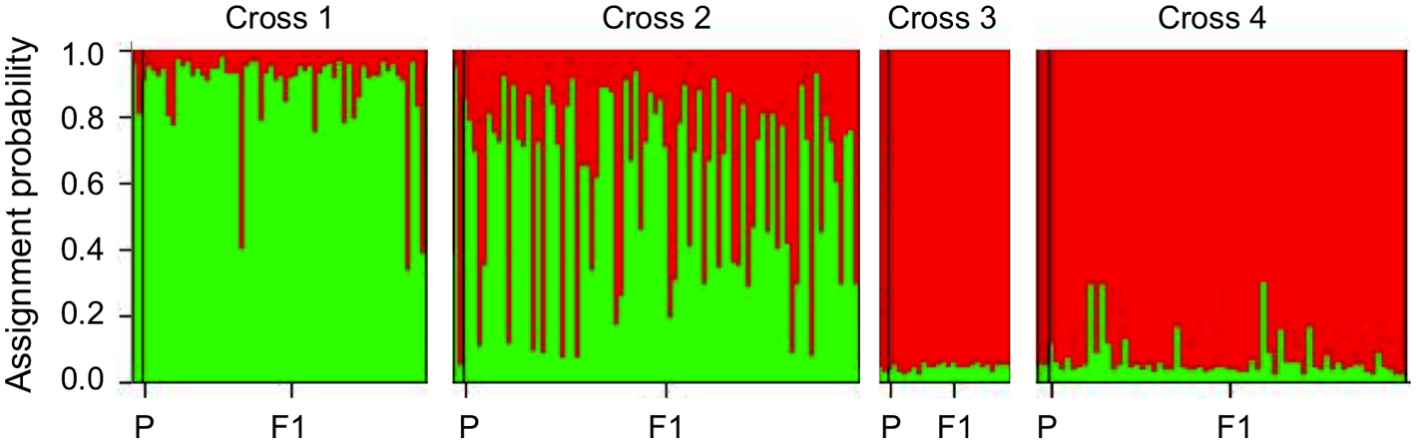
Population genetic structure of *Pseudo-nitzschia multistriata* parental strains and samples of their F1 offspring [21] as identified by Structure. For each cross, parental and F1-strains are grouped in boxes, ordered along the x-axis. Each vertical bar in a box represents one strain; the first two bars represent the parental strains, the remainder, the F1 strains. The y-axis indicates the proportion of a strain’s genome assigned by Structure to POP_A (green) and POP_B (red). Strains not assignable to one of these populations at or above the 0.9-probability-level are considered putative hybrids based on the Structure results. Parental strain assignment according to Structure: Cross 1: SY017 (POP_A; green)×SY278 (hybrid); Cross 2: SY017 (POP_A; green)×SY138 (POP_B; red); Cross 3: SY138 (POP_B; red)×SY378 (POP_B; red); and Cross 4: SY138 (POP_B; red)×SY379 (POP_B; red).

A Bayesian clustering analysis was carried out on the genotypes of the 525 strains from the field and the F1 strains generated from crossing experiments conducted in the study of Tesson *et al.* [11]. A cross between parental strains SY017 (POP_A) and SY278 (hybrid; cross 1 in **Fig. 7**, **S2 Table**) generated an F1 in which most strains were assigned to POP_A and a minority were classified as putative hybrids. A cross between parents SY017 (POP_A) and SY138 (POP_B; cross 2 in **Fig. 7**, **S2 Table**) generated an F1 in which most strains were classified as putative hybrids (i.e. 82.7% of F1). Crosses between parents SY138 (POP_B) and SY378 (POP_B) and parents SY138 (POP_B) and SY379 (POP_B; crosses 3 and 4 in **Fig. 7**, **S2 Table**) generated an F1 of which most strains were classified as POP_B and only a few as hybrids (i.e. 0 out of 25 F1 and 7 out of 62 F1 in crosses 3 and 4, respectively).

## Discussion

Results of the Bayesian analysis and the FCA of the microsatellite genotypes obtained from the *Pseudo-nitzschia multistriata* strains uncovered two genetically distinct populations (denoted POP_A and POP_B) in the Gulf of Naples. A substantial proportion of strains could not be assigned with a ≥90%-probability to either of these populations and may represent hybrids between them. POP_A dominated in 2008, was largely replaced by POP_B in 2009 and 2010, and apparently returned to dominance in 2011. This latter observation is based on only a single sample with 13 strains, but all of these are assigned to POP_A. Samples dominated by strains assigned to one population usually included a minority of strains assigned to the other, as well as strains classified as putative hybrids, suggesting that the two populations persisted in sympatry throughout the four years of our study. Crosses between parents assigned to the two populations produced viable progeny with a genetic fingerprint by and large comparable to that of field strains classified by Structure as putative hybrids.

### Genotypically distinct populations occur in sympatry

Genetically distinct populations in the same or overlapping areas, i.e., in sympatry or parapatry, have been reported before in planktonic species of diatoms (*Pseudo-nitzschia pungens, Ditylum brightwellii, Skeletonema marinoi*) [3], [6], [36], [37], [8] and dinoflagellates (*Alexandrium fundyense* and *A. minutum*) [9], [10], [38]. Our observation of two apparently sympatric populations among the Neapolitan strains of *P. multistriata* resembles results by Casteleyn *et al.* [3], who observed genotypically distinct populations within one of the three clades of *Pseudo-nitzschia pungens* co-occurring at their western European sample sites. Their sympatric populations exhibited an F_ST_ value comparable to that between the two Neapolitan *P. multistriata* populations. Remarkably high genotypic difference was detected between populations of *P. pungens* along the Pacific Northwest (F_ST_ = 0.418; [6]), but these populations were attributed subsequently to different clades and are likely to belong to different cryptic species [36]. Similarly, four genetically distinct populations detected in *Ditylum brightwellii* [37] probably also represented closely related species [39]. In our case, results demonstrate that strains of the two *P. multistriata* populations can interbreed, and therefore, belong to a single biological species.

When genetically distinct populations are detected within a species, environmental or biological factors can usually be identified that keep these populations apart. For instance, a population of the diatom *Skeletonema marinoi* in a Swedish fjord differed genetically from one in the nearby open sea (F_ST_ ranging from 0.200 to 0.267) [8]. In this case, a shallow sill limits water exchange between the fjord and the open sea, maintaining environmental differentiation [8]. In addition, the species produces resting spores that can survive in the sediments for decades [18], thus permitting these populations to persist in their distribution areas [8], [18]. Similarly, populations of the cyst-forming dinoflagellate *Alexandrium fundyense* from the open Gulf of Maine differed genetically from those in adjacent coastal ponds [10], although genetic differentiation was also recorded along the development of a single bloom [38]. In the case of *P. multistriata,* however, the two populations co-existed in a coastal area without physical barriers. Resting stages are not known for this species [40], nor do we have any evidence for gradual seasonal succession of POP_A and POP_B, thus excluding such explanations for the co-occurrence of genetically distinct populations in the Gulf of Naples.

Both *P. multistriata* populations appeared to persist over the entire growth season and throughout the four years of our study. Their relative proportions changed between consecutive years, but remained, by and large, similar across the growth season within years. The Gulf of Naples is an open system, exhibiting complex surface hydrodynamics and marked exchange with adjacent gulfs along the southwestern Italian coast and with the open Tyrrhenian Sea [41]. So, the detection of similar proportions between POP_A and POP_B throughout a growth season, or even between two consecutive growth seasons, leads us to conclude that these populations co-occur in the same proportions far beyond our sampling point.

### Population structure may change in consecutive years

This study is the first in *Pseudo-nitzschia* - or in any phytoplankton species as far as we are aware of - revealing a turnover of populations between consecutive years, i.e., the dominant population becoming a minority and *vice versa*. Apparently, the turnover happens when the species is not observed in the plankton. Since benthic resting stages are unknown for *Pseudo-nitzschia* [40], the species must persist in the plankton outside the growth season either below the detection threshold for routine LM-observation or beyond the depth range at which we sample the plankton.

Periods of extremely low densities could render populations prone to bottleneck effects. However, no such a bottleneck was evident from the results of the Wilcoxon tests. The genetic difference between POP_A in 2008 and POP_B in 2009 cannot be explained assuming a bottleneck since the alleles present in POP_B (2009 and 2010) do not represent a sub-set of the alleles present in POP_A (2008); POP_B exhibits several alleles absent in POP_A (2008). Therefore, other factors must be responsible for the turnover, possibly ones related to the life cycle or to mortality, affecting the populations differently.

### Possible effects of the life cycle on population structure

The virtual disappearance of POP_A after 2008 could be a consequence of the episodic nature of sexual reproduction in *P. multistriata* and a differential success of sexual reproduction in the two populations. We do not have direct observations of the sexual phase of this species in the field. Yet, a model developed by D’Alelio *et al.* [21] from patterns of cell size distribution in field samples over time predicts that sexual reproduction occurs in the autumn of every second year. This peculiar timing is made possible because cell size in *P. multistriata*, as in most other diatom species, is a function of time due to a gradual reduction of average cell size (length) with on-going mitotic division. Once the size-range for sexual maturity has been reached (ca. 55 µm, after two years in *P. multistriata*), cells can reproduce sexually, giving rise to large F1 cells (82–72 µm) [22].

The model predicts that towards the end of the growth season of every odd calendar year, all cells in the populations belong to a single, two-years old cohort of short cells (55–40 µm) that have reached the size-range for sexual maturity and can give rise to an F1 cohort of very long, juvenile cells towards the end of the growth season. The following growth season (i.e., in an even calendar year) shows a bimodal cell size distribution with short cells (ca. 50–30 µm) belonging to a now three years old remainder of the parental cohort, and a juvenile, one-year old cohort of long cells (70–55 µm) that are not yet sexually mature. The latter will reach sexual maturity only at the end of the next growth season (i.e., an odd year; see figure 3 in [21]).

The model [21] was inferred from cell size distributions observed in the field until 2006, whereas our observations span the years 2008–2011. If the model is extrapolated to our sample period, then only one cohort is expected during the growth seasons of 2009 and 2011, with sex occurring towards the end of these seasons, and two cohorts are expected in the years 2008 and 2010 without sex. The distribution of cell lengths in the natural samples (grey dots in **Fig. 3**) was indeed bi-modal in 2008. In 2009, the distribution was narrow and unimodal, representing the now sexually mature, two years old cohort. In 2010, the distribution was continuous but very broad, representing in the lower part the short cells belonging to the remainder of the 2007 cohort and in the upper part the long cells of the juvenile 2009 cohort.

In 2008, the cells sampled for genetic analyses (triangles in **Fig. 3**) belonged basically all to the remainder of the cohort that underwent sex in 2007 and was destined to perish between the growth seasons of 2008 and 2009. The vast majority of this cohort belonged to POP_A (green triangles, **Fig. 3**). In 2009, the sampled cells belonged to a single, now sexually mature cohort, with the vast majority of cells being assigned to POP_B (red triangles, **Fig. 3**) or classified as putative hybrids (black triangles, **Fig. 3**). In 2010, POP_B and putative hybrids dominated again, both among the three-years old remainder of the 2007-cohort of short cells as well as among its offspring - the one-year old 2009-cohort of long cells.

The virtual disappearance of POP_A between 2008 and 2009 could be due to a low success of sexual reproduction within POP_A versus that within POP_B at the end of the growth season of 2007. If this is correct, then we expect the larger cells in 2008 to belong mainly to POP_B. Unfortunately, we did not sample this cohort well; only three cells sampled in 2008 were long. The dominance of POP_B in both 2009 and 2010 is in accordance with expectations, because in 2009 a sexually mature cohort dominated by POP_B and hybrid cells must have generated a new cohort of large cells detected in the following year, belonging mainly to POP_B or classified as putative hybrids.

Thus, different success of sexual reproduction of the two populations in 2007 and a comparable success in 2009 could explain the observed patterns until the end of 2010. However, this explanation requires that the two populations use different cues or triggers to commence sexual reproduction, or that they have a mating preference for partners belonging to the same population, for neither of which we have any evidence. Moreover, a different reproductive success cannot explain the dominance of POP_A in the only sample available to us in 2011 and neither does it explain the marked rise in the proportion of POP_A at the end of the 2010 growth season. Therefore other factors must be at work to explain these transitions.

### Persistence of genetic structure in the face of hybridization

The persistence of POP_A and POP_B in large proportions over the four years is unexpected in the face of the large proportion of strains classified as putative hybrids and the apparent lack of reproductive barriers between POP_A and POP_B strains. At least under controlled laboratory conditions, cells of the opposite mating type undergo sexual reproduction and their F1 exhibit microsatellite genotypes largely in accordance with Mendelian inheritance rules [11]. Most F1 strains resulting from crossing POP_A and POP_B cells (cross 2) are not assigned to either one of these populations above the 90% probability threshold by Structure and are therefore classified as putative hybrids, suggesting that field strains not assigned to either one of these populations above the 90% probability threshold are hybrids in a biological sense as well. Such hybrids are fertile because a cross of a strain classified as hybrid with a POP_A-strain (cross 1) generated a perfectly viable F1, which in its turn is fertile as well (unpublished results). Results of the mating experiments in the lab show no evidence for mating barriers between the populations, though in these mating experiments partners were not offered a choice.

Our results indicate that hybridization is common between POP_A and POP_B also in the field. First, the results of the FCA reveal a continuum from POP_A *via* the assigned hybrids to POP_B, and even if the assigned hybrids are ignored, there exists marked gene flow between POP_A and POP_B. Second, many strains identified as putative hybrids by Structure shared alleles seen otherwise only among POP_A-strains with those observed otherwise exclusively among POP_B-strains. Third, samples composed of strains of two reproductively isolated populations should reveal a Wahlund effect, i.e., a lower number of heterozygotes than expected if the samples were composed of a single population in Hardy-Weinberg equilibrium [42]. Instead, almost all samples show heterozygote over-dominance. Such a pattern is typical for populations connected by substantial gene flow [42].

Interestingly, some F1-strains of cross 1 (hybrid×POP_A) were classified as putative hybrids and others as belonging to POP_A, indicating that a strain assigned to POP_A does not necessarily need to be derived from POP_A parents. Likewise, several F1-strains resulting from a cross between two POP_B parents were classified as putative hybrids. Two explanations can be given for these observations. First, assignment probability of a strain to, e.g., POP_B depends on the presence of private alleles for POP_B and on alleles found with higher frequency in POP_B strains. If an F1 strain happens to inherit from its POP_B parents alleles not very specific for POP_B, then its assignment probability to POP_B can fall below the 90% and hence, it is classified as a hybrid. Second, the precision of assignment probability improves with the number of loci, the number of individuals, and the F_ST_ between the two populations. The smaller these values, the larger the imprecision around an assignment. In the case of our data, the total number of strains is high but the number of loci and the F_ST_ are modest, suggesting that some of the assignments could be inaccurate. This argument does not explain why in the face of hybridization the majority of the strains remain assignable to POP_A or POP_B throughout the years of our sample campaign.

A reason why the two populations remain to be encountered in sympatry as genetically distinct entities in the face of hybridization might be that they both are relatively new arrivals. However, the species appeared in the Gulf of Naples in 1996 and must have gone since then through eight periods of sexual reproduction until 2011, according to the model by D’Alelio *et al.* [21]. If the two populations arrived simultaneously and remained confined in the Gulf of Naples ever since, then these eight phases of sexual reproduction sufficed to merge them entirely. However, this has not happened.

### Towards a meta-population explanation

Periodical appearance and disappearance of populations have been observed in meta-population structures of organisms living in fragmented habitats, where single local populations crash and the site is re-populated by other, distinct, but not completely disjoint, populations [43]. Apparently, *P. multistriata* could follow such a meta-population-like structure, with distinct but connected populations blooming in different regions. This scenario could explain away the sudden rise in the proportion of POP_A strains sampled at the end of the 2010 growth season, or any other change in the proportions. We lack information about the population genetic structure of *P. multistriata* in other coastal regions in the Mediterranean Sea and in other basins, though the species is known to occur elsewhere along the Tyrrhenian coastline [44] and is, in fact, distributed globally (see references in [45]). Casteleyn *et al*. [3] demonstrated the existence of multiple genetically distinct, geographically distant populations in *Pseudo-nitzschia pungens* clade I, with samples taken at each of the geographically distant locations containing one or a few immigrants, or members of minor resident populations, exhibiting genotypes typical for populations dominating elsewhere. Our results show that within the PCA plot in **Fig. 6**, the 2011-sample dominated by POP_A strains was recovered distantly from the cluster of 2008 samples dominated by POP_A, indicating genetic changes over time, possibly resulting from exchange with yet unknown populations elsewhere. Extensive genotyping of *P. multistriata* samples from geographically distant places will reveal if such a patchwork of populations exists also in *P. multistriata*.

In conclusion, we hypothesize that *P. multistriata* population dynamics in the Gulf of Naples follows a meta-population-like model, which includes establishment of populations by cell inocula from previous populations at the beginning of each growth season as well as remixing and dispersal governed by water masses that host these populations. Further multiannual studies of population genetic diversity and structure will help clarifying the environmental and internal factors that govern the evolution and fate of the populations of unicellular microalgae.

## Supporting Information

S1 Figure **The number of clusters (K; populations) as estimated following Evanno** ***et al.*** **** [29] **using the web-based program Structure Harvester** [30] **.** Independent runs were performed for K = 1 to 22; results for K>5 have been pruned from the figure.
(TIF)

S1 Table **Genetic parameters of the seven microsatellite loci of** ***Pseudo-nitzschia multistriata*** **.**
(DOCX)

S2 Table **Microsatellite multilocus genotypes of** ***Pseudo-nitzschia multistriata*** **strains, including parental and F1 strains from crossing experiments.** Footnotes for S2 Table: First column: code of 759 strains tested including 525 strains sampled at LTER-MC research station (SYxxx isolated in 2008–2010, and esxxx and mmxxx isolated in 2011), the two parental and the F1 strains isolated from the four crossing experiments. Second column: 1–22: the 22 sampling dates (Table 1) during which *P. multistriata* were isolated; 23, 25, 27, 29: the parental strains used in each crossing-experiment (SY017×SY278, SY017×SY138, SY138×SY378 and SY138×SY 379, respectively) and 24, 26, 28, 30: the four respective crossing-experiments. Third column: 1–3: genetic population (POP) as assigned by Structure software (1: POP_A, 2: POP_B and 3: putative Hybrids). Columns 4–17: allele size at each of the seven loci tested.
(XLSX)

S1 Data **Matrix_GenAlex_MC_POP.txt.** Matrix_GenAlex_MC_POP.txt has to be opened in Excel. The file is the input matrix that has been utilized for running the software GenAlex on Excel. It contains the microsatellite multilocus genotypes for the diatom species *Pseudo-nitzschia multistriata*, sampled at the LTER-MC research station in the Gulf of Naples, Italy, in 22 sampling dates along years 2008, 2009, 2010 and 2011. First row: the number of microsatellite loci amplified; the total number of strains; the number of samples and the number of strains in each sample. Second row: name of the analysis. Third row: names of the seven microsatellite loci amplified. First column: code of 525 strains tested and sampled at LTER-MC research station (SYxxx for 2008–2010 strains and esxxx and mmxxx for 2011 strains). Second column: 1–22: the 22 sampling dates during which *P. multistriata* were isolated. Third column: 1–3: the genetic population as assigned by Structure software (1: POP_A, 2: POP_B and 3: putative Hybrids). Columns 4–18: allele size at each of the seven loci tested.
(TXT)

S2 Data **Matrix_Structure_plot_MCPF1.txt.** Matrix_Structure_plot_MCPF1.txt is the input matrix that has been utilized for running the software STRUCTURE. Results are shown in Fig. 7. It contains the microsatellite multilocus genotypes for the diatom species *Pseudo-nitzschia multistriata*, sampled at the LTER-MC research station in the Gulf of Naples, Italy, in 22 sampling dates along years 2008, 2009, 2010 and 2011, the parents utilized in four crossing experiments and the relative F1 strains obtained. First row: name of the seven microsatellite loci amplified. First column: code of 759 strains tested: 525 strains sampled at LTER-MC research station (SYxxx isolated in 2008–2010, and esxxx and mmxxx isolated in 2011), the two parental and the F1 strains isolated from the four crossing experiments. Second column: 1–22: the 22 sampling dates during which *P. multistriata* were isolated; 23, 25, 27, 29: the parental strains used in each crossing-experiment (SY017×SY278, SY017×SY138, SY138×SY378 and SY138×SY 379, respectively) and 24, 26, 28, 30: the four respective crossing-experiments. Columns 4–17: allele size at each of the seven loci tested.
(TXT)

S3 Data **Matrix_Structure_qplot_MC1-22.txt.** Matrix_Structure_qplot_MC1-22.txt is the input matrix that has been utilized for running the software STRUCTURE. Results are shown in Fig. 4. It contains the microsatellite multilocus genotypes for the diatom species *Pseudo-nitzschia multistriata*, sampled at the LTER-MC research station in the Gulf of Naples, Italy, in 22 sampling dates along years 2008, 2009, 2010 and 2011. First row: names of the seven microsatellite loci amplified. First column: code of 525 strains tested and sampled at LTER-MC research station (SYxxx isolated in 2008–2010 and esxxx and mmxxx isolated in 2011). Second column: 1–22: the 22 sampling dates during which *P. multistriata* were isolated. Columns 4–17: allele size at each of the seven loci tested.
(TXT)
