# Supplementary figures and images for: Temporal Changes in Population Structure of a Marine Planktonic Diatom
Source: PLoS One. 2014 Dec 15;9(12):e114984. doi: 10.1371/journal.pone.0114984 (PMC4266644; doi:10.1371/journal.pone.0114984)

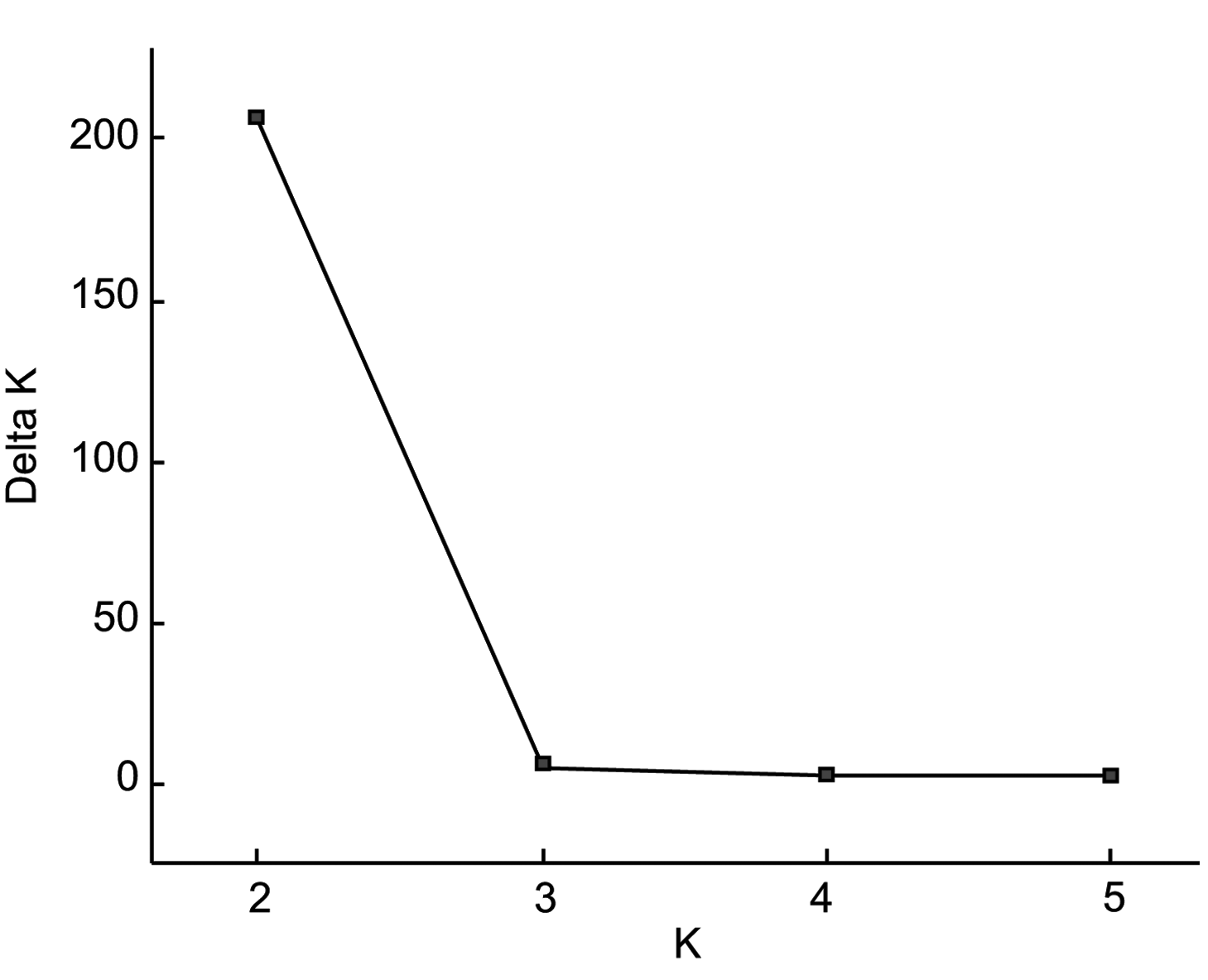

Supplement: S1 Figure — The number of clusters (K; populations) as estimated following Evanno et al. [29] using the web-based program Structure Harvester [30] . Independent runs were performed for K = 1 to 22; results for K>5 have been pruned from the figure. (TIF) [file pone.0114984.s001.tif]
