# Supplementary material for: Temporal Changes in Population Structure of a Marine Planktonic Diatom
Source: PLoS One. 2014 Dec 15;9(12):e114984. doi: 10.1371/journal.pone.0114984 (PMC4266644; doi:10.1371/journal.pone.0114984)
Supplement: S1 Table — Genetic parameters of the seven microsatellite loci of Pseudo-nitzschia multistriata . (DOCX) [file pone.0114984.s002.docx]

**Table S1**. Genetic parameters of the seven microsatellite loci of *Pseudo-nitzschia multistriata*.

| Locus | **N** | **Na** | **Range** | **He** | **Ho** | **NA** | **r** | **Stuttering** | **AD** |
| --- | --- | --- | --- | --- | --- | --- | --- | --- | --- |
| *PNm1* | 525 | 21 | 107-169 | 0.639 | 0.637 | YES | 0.0693 | YES | NO |
| *PNm2* | 525 | 10 | 167-197 | 0.397 | 0.491 | NO | -0.0236 | NO | NO |
| *PNm3* | 525 | 7 | 202-235 | 0.347 | 0.419 | NO | 0.0191 | NO | NO |
| *PNm5* | 525 | 14 | 218-268 | 0.528 | 0.471 | YES | 0.1013 | YES | NO |
| *PNm6* | 525 | 16 | 229-275 | 0.712 | 0.787 | NO | 0.0148 | NO | NO |
| *PNm7* | 525 | 9 | 250-292 | 0.502 | 0.644 | NO | -0.0711 | NO | NO |
| *PNm16* | 525 | 8 | 301-340 | 0.605 | 0.889 | NO | -0.1382 | NO | NO |

The total number of loci tested (N), the number of alleles (Na), the allelic size range (Range), the expected and observed heterozygosity (He and Ho), the presence of ‘null-alleles’ (NA) and their frequency (r), according to the Brookfield 1 ‘estimator’, the presence of scoring errors (Stuttering) and of large allele drop out (AD).
